# Dual challenges: endocarditis in a rare case of unicuspid aortic valve and double orifice mitral valve

**DOI:** 10.1093/ehjcr/ytaf329

**Published:** 2025-07-22

**Authors:** Mahboubeh Pazoki, Leila Nojoomizadeh, Seyede Saba Mostafavi Montazeri, Soroush Mostafavi

**Affiliations:** Department of Cardiology, Hazrat-e Rasool General Hospital, School of Medicine, Iran University of Medical Sciences (IUMS), Tehran, Iran; Department of Cardiology, Hazrat-e Rasool General Hospital, School of Medicine, Iran University of Medical Sciences (IUMS), Tehran, Iran; Student Research Committee, Alborz University of Medical Sciences, Karaj, Iran; Department of Cardiology, Hazrat-e Rasool General Hospital, School of Medicine, Iran University of Medical Sciences (IUMS), Tehran, Iran

## Case description

**Figure 1:**
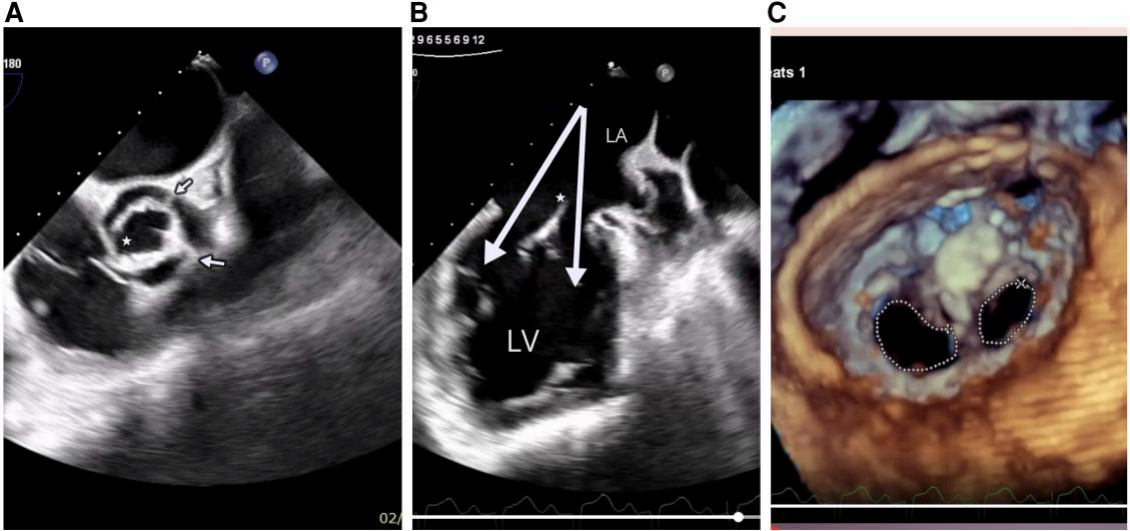
*A*) Unicuspid unicommisural aortic valve with two raphes (arrows) and one commissure (star). *B*) Transoesophageal echocardiography showed double orifice mitral valve (arrows) with fibrous bridge (star). *C*) Asymmetrical double orifice mitral valve 3D reconstruction.

A 45-year-old IV drug user (methamphetamine) was referred to our hospital with a complaint of prolonged fever and weakness for over 1 month. He had a history of coarctation of aorta that underwent surgery 15 years ago. At that time, surgery on the valves was deemed unnecessary due to the absence of severe regurgitation or stenosis. Auscultation showed a significant early diastolic murmur (Levine V/VI), heard best at the third left intercostal space. First laboratory data were normal white blood cell (WBC) count (8000/MM^3^), increased C-reactive protein level (54 mg/L), and 48 h blood cultures were done for the patient. Evaluation for hepatitis B, C, human immunodeficiency virus, and Brucella returned negative. Empiric antibiotic therapy was started with ceftriaxone and vancomycin. The presence of suspicious mobile vegetation on unicuspid aortic valve (UAV) in transthoracic echocardiography was noticed. No evidence of aortic stenosis was observed in the examination and echocardiography. The mitral valve was suspicious for a double orifice mitral valve (DOMV). The ascending aorta was dilated (3.8 cm). Consequently for better evaluation of valvular structures, a transoesophageal echocardiography was necessitated. Transoesophageal echocardiography revealed UAV (*Figure 1A*) with large hypermobile vegetation (length: 4 cm, thickness: 1 cm) on aortic valve, protruding to aorta and left ventricular outflow tract during systole and diastole (Supplementary material online, *Video 1*) resulting in severe aortic regurgitation with holodiastolic flow reversal in abdominal aorta (velocity time integral: 16 cm). Transoesophageal echocardiography confirmed asymmetrical DOMV (*Figure 1B* and Supplementary material online, *Video 2*) with progressive stenosis (one orifice area by 3D zoom (*Figure 1C*) method 1.7 cm^2^ and another one was 1 cm^2^) and moderate mitral regurgitation. These three left sided congenital obstructive heart defects (UAV, DOMV, and coarctation of the aorta) are considered part of the Shone’s complex.^1^ Meanwhile, the patient was a candidate for cardiac surgery due to a cardiac surgery consultation. But unfortunately before surgery, the patient passed away. The patient underwent cardiopulmonary resuscitation due to sudden loss of consciousness, which was probably due to cerebral embolism of valvular vegetations. Autopsy was not allowed to diagnose the definite cause of death. 48 h blood cultures were negative. The patient had no recent antibiotic use, but it is possible that he had endocarditis with culture-negative organisms.

## Supplementary Material

ytaf329_Supplementary_Data

## Data Availability

Data available on request.

## Supplementary material

**Consent:** The authors confirm that written consent for the submission and publication of this work has been obtained from the patient, in accordance with the COPE guidelines.

**Funding:** None declared.
